# Potential of Triterpenic Natural Compound Betulinic Acid for Neglected Tropical Diseases New Treatments

**DOI:** 10.3390/biomedicines10040831

**Published:** 2022-04-01

**Authors:** Vinícius Rocha, Helenita Quadros, Cássio Meira, Laís Silva, Dahara Carvalho, Katharine Hodel, Diogo Moreira, Milena Soares

**Affiliations:** 1 Institute of Health Technology (SENAI CIMATEC ITS), Salvador 41650-010, BA, Brazil; cassio.meira@fieb.org.br (C.M.); katharine.hodel@fieb.org.br (K.H.); milena.soares@fieb.org.br (M.S.); 2 Gonçalo Moniz Institute, Oswaldo Cruz Foundation (IGM-FIOCRUZ/BA), Salvador 40296-710, BA, Brazil; helenita_quadros@hotmail.com (H.Q.); laisperez1@hotmail.com (L.S.); daharacarvalho@gmail.com (D.C.); diogo.magalhaes@fiocruz.br (D.M.)

**Keywords:** leishmaniasis, Chagas disease, malaria, neglected tropical diseases, betulinic acid, treatment

## Abstract

Neglected tropical diseases are one of the most important public health problems in many countries around the world. Among them are leishmaniasis, Chagas disease, and malaria, which contribute to more than 250 million infections worldwide. There is no validated vaccine to prevent these infections and the treatments available are obsolete, highly toxic, and non-effective due to parasitic drug resistance. Additionally, there is a high incidence of these diseases, and they may require hospitalization, which is expensive to the public health systems. Therefore, there is an urgent need to develop new treatments to improve the management of infected people, control the spread of resistant strains, and reduce health costs. Betulinic acid (BA) is a triterpene natural product which has shown antiparasitic activity against *Leishmania*, *Trypanosoma cruzi,* and *Plasmodium*. Here, we review the main results regarding the in vitro and in vivo pharmacological activity of BA and its derivatives against these parasites. Some chemical modifications of BA have been shown to improve its activities against the parasites. Further improvement on studies of drug-derived, as well as structure–activity relationship, are necessary for the development of new betulinic acid-based treatments.

## 1. Introduction

Neglected tropical diseases (NTDs) represent a huge problem in many countries worldwide. NTDs are caused by a variety of pathogenic microorganisms, such as bacteria, fungi, viruses, and parasites. The World Health Organization (WHO) recognizes 20 major NTDs spread around the world. Approximately 2 billion people are affected, including 0.5 billion children, and 200,000 people die per year from NTDs. Together, NTDs are involved in the loss of 25.1 million disability-adjusted life years, 16.9 million years lived with disability, and 8.21 million years of life lost [1,2]. People living in countries located in the tropical and subtropical regions are the most affected by NTDs, especially because these diseases are also exacerbated by the poverty levels, low health assistance, and precarious basic sanitation. Nowadays, due to the COVID-19 pandemic, the impact of NTDs has significantly increased because funding is currently allocated predominantly for diagnostic, vaccines, and therapeutic research on COVID-19 [3,4,5,6]. Among the NTDs, the protozoan-borne diseases are the most prevalent and responsible for more than one million of deaths per year and one-sixth of the world population is at risk of infection. Among the NTDs spreading around the world, Leishmaniasis, Chagas disease, and Malaria contribute to a frightening number of deaths per year. Moreover, these NTDs impair economics of countries via loss of work and high treatment costs, along with psychologically affecting people [7].

Leishmaniasis is a series of diseases caused by more than 20 species of *Leishmania* parasites, which are transmitted by the bite of female phlebotomine sand flies. These diseases are estimated to be the ninth largest disease burden among individual infectious diseases [8]. Different clinical forms develop as a consequence of the parasite species associated with the infection and the immune status of the patient [9]. The visceral leishmaniasis presents high mortality mainly among infants. Moreover, the number of HIV/leishmania coinfections have increased around the world, mainly on the African continent [10]. The cutaneous clinical manifestation is not lethal but causes scar formation due to secondary infections and ulcerations, and disfiguring deformations when it evolves to the mucocutaneous form [11]. Regardless of the clinical form, *Leishmania* infections have a prevalence among 350 million people living in high-risk areas, with 12 million known cases and an incidence of around 2 million new cases per year [10].

Chagas disease, caused by *Trypanosoma cruzi* infection, represents an important public health problem, affecting around 7 million people worldwide. Classically, Chagas disease is transmitted to animals and people by hematophagous triatomine vectors [12]. However, the parasite can also infect the vertebrate host by other routes, such as blood transfusion, organ transplantation, laboratory accidents, congenital transmission, or accidental ingestion of crushed insects with food (oral contamination), such as sugarcane and guava juices, and açaí [13]. Although Chagas disease is endemic to Latin American countries, due to the intense migratory flow, an increase in the number of cases is now seen in non-endemic countries of North America, Europe, Asia, and Oceania, and, in particular, the United States, Canada, Spain, France, Switzerland, Japan, emerging Asian countries, and Australia [14]. Chagas disease has two phases: an acute phase, corresponding to the infection and dissemination of *T. cruzi* in the body, and a chronic phase, with different forms, which can be asymptomatic (indeterminate) and symptomatic. The acute phase is marked by high parasitemia and intense inflammatory response, leading to tissue damage which may affect the heart, liver, and spleen [15]. After the acute phase, about 20–40% of patients develop digestive form and/or chronic Chagas cardiomyopathy (CCC) months or decades after infection [12,13].

Malaria, a disease caused by *Plasmodium* parasites, is a major life-threatening disease in tropical and subtropical regions worldwide, leading to about half million deaths annually [16]. Among the 200 species of *Plasmodium* known to date, five of them, *P. falciparum, P. vivax*, *P. ovale*, *P. malariae*, and *P. knowlesi,* can infect humans [17]. Of these, *P. falciparum* causes the most serious infection and is considered the main species responsible for almost all cases and deaths [18]. According to the World Malaria Report 2021 by the World Health Organization (WHO), the burden of malaria cases has increased from 227 million cases in 2019 to 241 million in 2020. This alarming number of malaria cases is mostly located in the Sub-Saharan African countries, which comprise 95% of malaria cases, the majority of these affecting children under the age of five [19,20]. The current strategies developed for malaria prevention and vector control include the use of long-lasting insecticide-treated bed nets, early diagnosis and treatment with artemisinin-based combination therapies (ACTs), and chemoprevention in pregnant women and young children [21]. In addition, an effective malaria vaccine would be an important tool to be implemented for preventing *Plasmodium* infection due to its ability to induce a potent and protective immune response in phase 3 clinical trials with the first and most promising malaria vaccine RTS,S/AS01 (Mosquirix™) it had limited efficacy in residents of malaria-endemic regions and, thus, demonstrates the need for further evaluation before its use is adopted [22,23]. In the absence of an efficacious malaria vaccine, the therapeutic use of antimalarial drugs remains the only promising strategy for prophylaxis and treatment of malarial disease.

A common feature among leishmaniasis, Chagas disease, and malaria is the low efficacy of treatments due to parasite resistance and/or high toxicity. The drugs used to treat these NTDs are, in general, old, obsolete, toxic, non-compatible with patient compliance, and involved with parasitic drug resistance development.

The first-line drugs to treat leishmaniasis are pentavalent antimonials, discovered to be effective in the 1940s. This treatment is extremely toxic and should not be used in elderly people, persons with cardiac diseases, and pregnant women. The treatment causes excess mortality, even in cases of cutaneous leishmaniasis. Amphotericin B is the drug of choice in some countries where *Leishmania* is completely resistant to antimonial. Antimonial has high toxicity and is expensive to formulate into liposomes. Other medicines such as miltefosine, the only oral treatment, paramomycin, pentamidine, and alternative therapies, such as thermotherapy, can be used during the treatment of leishmaniasis. However, there is still a lack of effective and safe therapies along with vaccines for this NTD [24,25]. Despite more than 100 years of research, Chagas disease treatment is limited only to two drugs, benznidazole and nifurtimox. Both drugs are prodrugs and need to be activated by an enzyme known as trypanosome nitroreductase I. These drugs act through the formation of free radicals and/or electrophilic metabolites, which damage proteins, lipids, and the DNA of *T. cruzi* [26]. Benznidazole, due to its better efficacy and safety profile when compared to nifurtimox, is used as a first-line treatment [27]. Both drugs are recommended for the treatment of Chagas disease in the acute phase, congenital infection, as well as in cases of reactivation of the infection in transplanted patients [28]. However, they have low efficacy in the chronic phase of the disease and several side effects are associated with their use, such as skin rashes, nausea, kidney and liver failure, and peripheral neuropathy [28]. Therefore, new drugs for Chagas disease are still needed.

In the long past, malaria has been treated with natural products found in bark, roots, or leaves of plants; however, only in the last century have the active molecules been extracted, chemically modified, and used as isolated drug compounds for the treatment [29]. In fact, antimalarial drugs based on natural products, semi-synthetic, and synthetic compounds have been developed since the 1940s [30,31,32,33]. Quinine and artemisinin are examples of plant-derived potent antimalarial agents, whose synthetic derivatives also play a role in the chemotherapy [33]. The antimalarials in clinical use for prophylaxis and treatment, such as the 4-aminoquinolines chloroquine and amodiaquine, the aminoalcohols quinine and mefloquine, the 8-aminoquinolines primaquine and tafenoquine, and the endoperoxides artemisinin and their derivatives, present great efficacy, good availability, low toxicity, and affordable costs [30]. However, the exacerbated use of these drugs in a monotherapy strategy, together with a lack of effective control strategies to block the transmission of *Plasmodium* species, resulted in the appearance of resistance strains and, therefore, the impairment of the therapeutic efficacy. Thus, the high failure rates of monotherapy for malaria treatment led the WHO to recommend the use of two or more compounds with different modes of action to fight malarial infection efficiently [29,32]. Nevertheless, the development of new and effective antimalarials remains a global challenge.

In view of this scenario, there is an urgent need to develop new therapeutic options to treat these neglected infections. The sequencing of the genome of the causative parasites opened several opportunities for drug development, using validated drug targets to screen compounds [34,35]. However, pharmaceutical companies have low interest in research and development of new treatments for NTDs because of the expected low return on investments. Therefore, despite the technological advances in parasitology, chemistry, and genomics, there are not new medicines approved to treat these diseases. In this case, universities and public research institutes are the main players focused on drug discovery and development against these NTDs [8]. In this scenario, alternative treatment options have emerged, including drug repurposing [8], cell therapy [36], and even antiparasitic phage therapy [37], approaches which still need validation by robust clinical trials and authorization by regulatory agencies.

Phenotypic screening is the most applied methodology to screen extracts, fractions, and purified compounds against these parasites. Indeed, plant biodiversity represents a promising source of active biomolecules to treat NTDs [38]. Natural products are used as a source of active molecules, as well a source of structure models to design derivative molecules. An interesting example is betulinic acid (BA), a lupane-type triterpene present in *Betula* species and many other plants [39]. BA has been associated with a wide range of pharmacological effects, including anti-cancer, antioxidant, anti-inflammatory, antiatherosclerotic, antiviral, hepatoprotective, and immunomodulatory activities [40]. Several studies have shown that this secondary metabolite and its derivatives have demonstrated pharmacological activity against different parasites. In this work, we review the main results of BA in preclinical studies in leishmaniasis, Chagas disease, and malaria models. Our aim is to compilate these data and show that BA and its derivatives deserve more deep studies to understand their pharmacodynamics, followed by the clinical development of the drug as an alternative treatment for these parasitic diseases.

## 2. Antiparasitic Activity of Betulinic Acid and Its Derivatives 

### 2.1. Anti-Leishmania Activity of Betulinic Acid

The anti-*Leishmania* activity of BA and derivatives have been determined and Table 1 summarizes the main activity values of BA present in plant extracts, and as a pure compound, as well as its derivatives tested. BA was found to be one of the biomolecules isolated from *Pelliciera rhizophorae,* which is an endemic plant from mangroves. Among the biomolecules isolated from this plant, BA was assayed against *L. donovani* axenic amastigotes. However, this compound did not present significant activity either against *Leishmania* or *T. cruzi*. Since BA presented an inhibitory concentration of 50% (IC_50_) equal to 18 μM against *P. falciparum*, this suggests the absence or a redundant function of a common drug target in trypanosomatid parasites to explain the absence of activity in trypanosomatids (Table 1) [41]. Similar data were reported by Mamdouh and colleagues (2014), showing that BA isolated from *Syzygium samarangense* did not present activity against *L. major* [42]. The BA was also isolated from the dichloromethane fraction from *Millettia richardiana* barks. This fraction was active against *Leishmania*, presenting an IC_50_ 10-fold higher than the reference drugs (11.8 µg/mL versus 1.4 µg/mL for pentamidine and 1.2 µg/mL for miltefosine), considering it is an extract compared with a purified drug. However, the BA isolated from this fraction showed moderated activity against *L. major* and an IC_50_ of 40 μM (Table 1) [42]. These data suggest that BA, found in many plants which are popularly used based on ethnopharmacological knowledge, presents low activity against *Leishmania* promastigotes and amastigotes. Therefore, different derivative molecules were synthesized with the aim to improve the anti-*Leishmania* activity.

Some BA derivatives produced and tested against *Leishmania* presented lower IC_50_ values, suggesting an improvement of its pharmacological effect. The first study of BA derivatives against *Leishmania* showed that the dihydrobetulinic acid was able to inhibit the interaction between topoisomerases I and II with the parasite DNA, inducing DNA brake and an apoptotic-like death in promastigotes and amastigotes. The IC_50_ values were significantly lower than that of BA: 2.6 and 4.1 μM for *L. donovani* promastigotes and amastigotes, respectively [45]. Moreover, dihydrobetulinic acid decreased 92% of the *L. donovani* infection in golden hamsters when administrated at 10 mg/kg body weight for 6 weeks [45]. On the other hand, other BA derivatives did not present promising results compared to dihydrobetulinic acid, in terms of anti-*Leishmania* activity and host cell toxicity. This is the case of betulonic acid, betulin 3-caffeate, BA, and betulin aldehyde (Table 1) [46,47,48,49].

The semisynthetic lupane triterpenoids betulin and BA derivatives were developed and assayed against *L. infantum* promastigotes. Out of sixteen compounds generated, two presented selective cytotoxicity to the parasite, the triterpenoid betulin, and one BA derivative. The compounds 3b-Hydroxy-(20R)-lupan-29-oxo-28-yl-1H-imidazole-1-carboxylate (BT06) and 28-(1H-imidazole-1-yl)-3,28-dioxo-lup-1,20(29)-dien-2-yl-1H-imidazole-1-carboxylate (BT13) showed an IC_50_ of 50.8 and 25.8 μM, respectively (Table 1). The toxic effect to the parasite was associated with the G0/G1 phase cell cycle arrest, followed by a rounded morphological change, but it was not associated with a significant apoptosis/necrosis induction. This effect seemed to be selective to the parasite, since both drugs did not induce host cell cytotoxicity. Moreover, isobologram analysis showed a synergistic interaction between BT13 with miltefosine, reducing the IC_50_ from 25.8 to 6 μM [44]. It suggests that BT06 and BT13 may represent a promising structure for the development of potential hits compounds.

Among the heterocyclic betulin derivatives, heterocycloadduct between 3,28-di-O-acetyllupa-12,18-diene and 4-methylurazine presented the highest activity against *L. donovani* amastigotes. Based on that, 24 derivatives were designed by chemical modifications at positions C-3, C-28, and C-20–C-29 of the lupane skeleton. These derivatives were tested against axenic amastigotes and THP-1-infected human macrophages. The betulonic acid eliminated 98% of axenic amastigotes and 85.3% intracellular parasites at 50 μM (Table 1), and the carbon–carbon double bond seemed to be important for this activity [46]. Modifications in BA backbone can improve the anti-*Leishmania* activity. In general, these modifications include the C-3 hydroxyl group esterification or oxidation [53].

**Figure 1:**
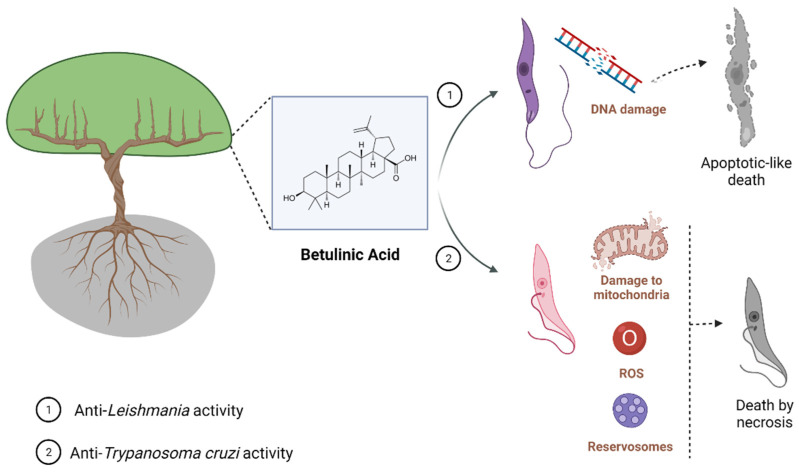
Known mechanisms of parasite activity against *Leishmania* and *T. cruzi***.** Previous work showed that dihydrobetulinic acid inhibits the interaction between topoisomerases I and II with the parasite DNA, inducing DNA brake and an apoptotic-like death in promastigotes and amastigotes. Regarding *T. cruzi*, it was shown a direct effect of BA5 on plasma membrane integrity, the formation of numerous and atypical vacuoles within the cytoplasm of the parasite, dilatation of some Golgi cisternae, and appearance of profiles of endoplasmatic reticulum involving organelles accompanied by the formation of autophagosomes, which ultimately result in trypomastigote cell death by necrosis. BA5 also reduced M1 markers and upregulated the M2 markers, inducing a regulatory phenotype.

BA has also been incorporated into nanoformulations containing nanochitosan which showed promising results against Leishmania. BA-containing nanoformulations at a concentration of 20 µg/mL were able to inhibit above 80% the growth of promastigote and amastigote forms of *L. major* and increased production of nitric oxide (NO), an important metabolite which contributes to anti-*Leishmania* activity of the host cell, in infected macrophages. A BA-containing nanoformulation was also tested in vivo, and at 20 mg/kg it reduced lesion size in *L. major*-infected mice (Table 1) [49,50]. A nanoformulation containing BA was also active against *L. donovani*-infected macrophages, and this activity correlated to the increase of NO e interleukin (IL)-12 and reduction of IL-10 production (Table 1) [51]. These results suggest that a carrier able to increase BA levels at the site of action may improve the activity of this compound in vivo through the direct action of BA against the parasite and, indirectly, by the immunomodulatory activity [40,54]. More studies regarding the use of drug carriers and BA are needed to explore in depth the benefic effects of BA and its derivatives. Moreover, it is well known that cutaneous and mucocutaneous leishmaniasis are characterized by high inflammatory infiltration, low parasite burden at the lesion site, and tissues necrosis [55,56]. Then, the use of a compound showing dual activity (anti-*Leishmania* and anti-inflammatory activities) will contribute to the treatment of the clinical manifestations of Leishmaniasis restricted to the skin. Figure 1 shows the main effect found, for now, related to parasite-killing by BA treatment.

Recently, our research group evaluated the leishmanicidal effect of BA5, a BA derivative, previously tested against *T. cruzi*. The BA5 presented activity against different species of *Leishmania* promastigotes with an IC_50_ of 4.5 ± 1.1 μM against *L. amazonensis*, 3.0 ± 0.8 μM against *L. major*, 0.9 ± 1.1 μM against *L. braziliensis*, and 0.15 ± 0.05 μM against *L. infantum*. This derivative also significantly reduced the percentage and parasitism in infected peritoneal macrophages without host cell toxicity, presenting an IC_50_ of 4.1 ± 0.7 μM against intracellular parasites. BA5 was able to induce membrane blebbing, flagella damage, and cell shape alterations in treated parasites. Moreover, BA5 acts synergistically to the amphotericin B-killing *Leishmania* parasite [52].

### 2.2. Anti-T. cruzi Activity of Betulinic Acid

The antiparasitic activity of BA has also been validated against *Trypanosoma cruzi*. Table 2 summarizes the main activity values of BA derived from extracts and as pure compounds. The first evidence that BA has trypanocidal activity was obtained in studies with extracts and fractions containing BA. Campos et al. (2005) evaluated the anti-*T. cruzi* activity of extracts and fractions from the plant *Bertholletia excelsa* against trypomastigotes forms (Y strain) [57]. A significant trypanocidal activity of acetone and methanol extract was observed at 500 µg/mL, which promoted a reduction of 100% and 90.3%, respectively, in trypomastigote viability. Furthermore, BA purified from the hexane extract inhibited 75.4% trypomastigotes viability at 500 µg/mL [57]. Extracts and fractions of *Ampelozizyphus amazonicus Ducke* (Rhamnaceae), a native tree from the Amazon forest, also showed trypanocidal activity against trypomastigotes forms (Y strain) and proved to be a source of bioactive compounds, including BA (Table 2) [58].

Most of the subsequent investigations about the trypanocidal effect of BA were carried through in vitro experiments, using Y or Tulahuen *T. cruzi* strains. Interestingly, BA presented anti-*T. cruzi* activity against all evolutive forms of *T. cruzi*. Dominguez-Carmona et al. (2010) showed BA trypanocidal activity against epimastigotes (Tulahuen strain), with an IC_50_ value of 50 µg/mL [53]. Cretton et al. (2015) demonstrated the activity of BA against *T. cruzi* amastigote forms (Tulahuen strain), with an IC_50_ value of 24.16 µg/mL (Table 2) [59]. In addition, Sousa et al. (2017) showed the inhibitory effect of BA on the growth of epimastigotes after 24, 48, and 72 h of incubation with IC_50_ values of 73.43, 119.8 μM, and 212.2 μM, respectively, in trypomastigotes with IC_50_ values of 51.88 μM after 24 h of incubation, and in amastigotes with IC_50_ values of 25.94 μM after 24 or 48 h of incubation (Table 2) [60]. In addition, the mechanism of parasite death was also investigated in epimastigotes forms, indicating that BA promotes alterations in the mitochondrial membrane, increase in reactive oxygen species, and swelling in reservosomes, which leads to parasite cell death by necrosis [60].

Lastly, in order to explore the possibility of improving the trypanocidal activity of BA, semisynthetic derivatives were prepared and evaluated against *T. cruzi*. Meira et al. (2016) screened a series of amide semisynthetic derivatives of BA and identified the derivative BA5 as a promising trypanocidal agent [61]. BA5 showed a potent anti-*T. cruzi* activity, with values of IC_50_ against amastigotes (IC_50_ = 10.6 µM) and trypomastigotes (IC_50_ = 1.8 µM) lower than benznidazole (IC_50_ amastigotes = 13.5 µM; IC_50_ trypomastigotes = 11.4 µM) (Table 2) [61]. Interestingly, BA5 also demonstrated a potent trypanocidal effect against amastigote forms in an infection model using human cardiomyocytes derived from induced pluripotency stem cells, showing an IC_50_ value of 3.2 µM, whereas, under the same conditions, benznidazole had an IC_50_ value of 5.9 µM [62]. Using transmission electron microscopy, it was possible to observe a direct effect of BA5 on plasma membrane integrity, the formation of numerous and atypical vacuoles within the cytoplasm, dilatation of some Golgi cisternae, and appearance of profiles of endoplasmatic reticulum involving organelles accompanied by the formation of autophagosomes, which ultimately result in trypomastigote cell death by necrosis. Most importantly, BA5 combined with benznidazole exhibited synergistic activity against trypomastigotes and amastigotes, which is an interesting finding due to the fact that drug combinations are being largely employed to combat parasitic diseases [44,63,64].

Finally, in a mouse model of chronic Chagas disease, BA5 (at 10 or 1 mg/Kg) decreased inflammation and fibrosis in the hearts of *T. cruzi*-infected mice. These effects were accompanied by a reduction of proinflammatory cytokines, such as interferon gamma, IL-1β, and tumor necrosis factor, and increased IL-10 production. Moreover, BA5 promoted an increase in the expression of macrophage M2 markers, such as arginase 1 and chitanase-3-like protein 1, and a decrease in M1 markers, such as nitric oxide synthase 2, which suggests a polarization to anti-inflammatory/M2 macrophage phenotype in mice treated with BA5 [65].

Altogether, these findings reinforce that BA has trypanocidal activity, and chemical modifications on BA skeleton might enhance trypanocidal activity. However, future investigations in animal models of acute and chronic *T. cruzi* infection need to be carried out in order to develop BA-based new treatments. Figure 1 shows the main effect found, for now, related to parasite-killing by BA treatment.

### 2.3. Anti-Plasmodium Activity of Betulinic Acid

The antiparasitic activity of BA has been also investigated against *Plasmodium* [66]. Bringmann et al. [67] demonstrated the in vitro antimalarial action of BA isolated, for the first time, from *Triphyophyllum peltatum* and *Ancistrocladus heyneanus*, against the *P. falciparum* asexual blood stages, achieving an IC_50_ value of 10.46 µg/mL against the chloroquine-sensitive strain NF54. Interestingly, BA isolated from a Tanzanian tree *Uapaca nitida* Müll-Arg. (Euphorbiaceae) was evaluated for its in vitro activity against T9-69 chloroquine-sensitive and K1 chloroquine-resistant *P. falciparum* strains. BA was found to have a similar low potency for both strains, with IC_50_ values of 19.6 µg/mL and 25.9 µg/mL, respectively (Table 3). Moreover, these authors assessed, for the first time, the in vivo activity of BA in a mouse model using *P. berghei*, but the dosage of 250 mg/kg resulted in animal toxicity and no parasitemia reduction [68]. After these first reports, more studies have been conducted to assess the antimalarial activity of BA and structurally related natural products.

In a study conducted by Suksamrarn and collaborators [69], 10 triterpenes isolated from *Gardenia saxatilis* were assessed in vitro against *P. falciparum* K1, a chloroquine-resistant strain. The results showed that 27-O-*p*-(Z)- and 27-O-*p*-(E)-coumarate esters of BA, and a mixture of uncarinic acid E (27-O-*p*-(E)-coumaroyloxyoleanolic acid) presented a good antiplasmodial activity with IC_50_ value of 3.8 µg/mL. Additionally, with similar potency, 27-O-*p*-(Z)- and 27-O-p-(E)-coumarate esters of BA, a mixture of uncarinic acid E (27-O-*p*-(E)-coumaroyloxyoleanolic acid) and 27-O-*p*-(E)-coumaroyloxyursolic acid, revealed an IC_50_ value of 2.9 µg/mL, while BA was not active at the same concentration range (IC_50_ ≥ 20 µg/mL). Therefore, the addition of a p-coumarate moiety at the 27-position may contribute to increasing the antimalarial properties of BA derivatives (Table 3).

**Figure 2:**
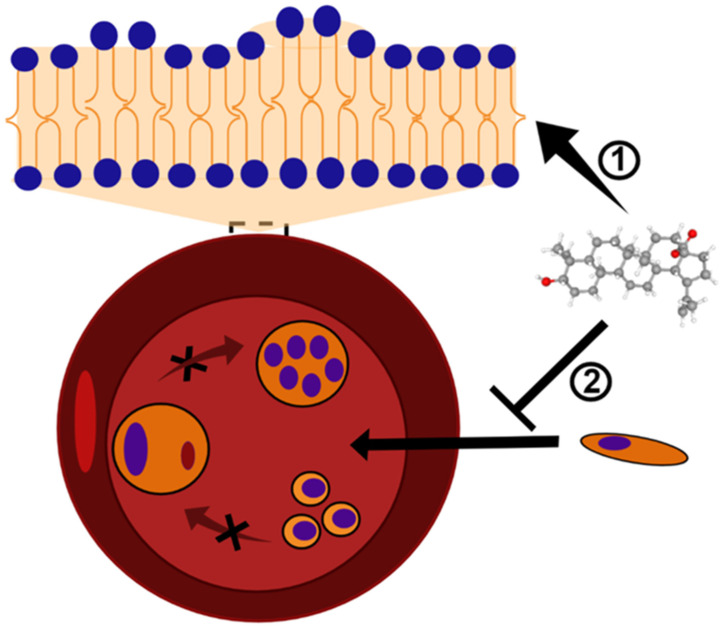
BA and analogs may cause deformation in the lipid bilayer and, consequently, alterations in the erythrocyte shape. This mechanism of action results in the formation of echinocytes or stomatocytes structures ➀, and inhibits the *Plasmodium* invasion and growth ➁. The black X symbol represents a blockage of *Plasmodium* maturation into erythrocytes. BA structure (CID 64971) was obtained from PubChem [81].

In the study by Ziegler et al. [75], BA and its analogs were evaluated in vitro for their ability to alter the erythrocyte shape and prevent *P. falciparum* invasion and growth. The BA analogs with a functional chemical group of donating a hydrogen bond caused the formation of echinocyte structures, whereas the analogs lacking this ability induced the formation of stomatocytes. The incorporation of the compounds into the lipid bilayer of erythrocytes may cause a membrane curvature alteration, which apparently presents an inhibitory role for *P. falciparum* invasion and further development (Figure 2). In agreement, a different study evaluated BA as well as lupeol, another pentacyclic triterpene, and also found that the incorporation of these acids in the host red blood cells consist of a mechanism inhibitory of parasite growth and development by preventing the merozoite internalization [75]. The triterpenoids, including BA, are known to reduce membrane fluidity, and this phenomenon is related to the interaction to the cholesterol-rich membrane rafts. Parasites die into the erythrocyte because its intercellular viability needs the vesicular traffic from the membrane to the vacuole-containing *Plasmodium*. The erythrocyte membrane modification caused the triterpenoids to affect the traffic of raft-anchored proteins of the erythrocyte host to the internalized *Plasmodium* vacuoles [76]. This effect of lupane triterpenoids has also been seen in other cell membranes and also mediates other pharmacological effects of BA, such as against malignant cells [77].

Other mechanisms of action for BA and derivatives in *Plasmodium* infection, such as inhibition of β-hematin formation and modulating calcium pathways in the parasite, have been also investigated [78], but it remains less clear the participation of these mechanisms for the antiparasitic activity of BA.

Other plants have been reported as sources of BA. For instance, Cui-Ying Ma and colleagues [70] produced a chloroform-extract from parts of *Diospyros quaesita* Thw. (Ebenaceae) and tested for in vitro antimalarial activity on cultures of *P. falciparum* clones D6 (chloroquine-sensitive) and W2 (chloroquine-resistant). The results showed an antimalarial activity with IC_50_ values of 8.1 µM and 8.3 µM, respectively (Table 3). In the search for active constituents, the extract went through a series of separations in which seven isolated compounds were generated, including BA. In corroboration with the abovementioned studies [67,68], BA also had a moderated antiplasmodial activity, with an IC_50_ value of 8.1 µM for D6 and 8.3 µM for W2 (Table 3), being approximately eight-fold lower than betulinic acid 3-caffeate and betulinic acid 3-diacetylcaffeate for both *P. falciparum* strains; however, differently to these two other compounds, BA did not show cytotoxicity for the cell type tested. Lenta et al. [71] isolated BA from hexane extracts of *Psorospermum glaberrimum* and evaluated in vitro against *P. falciparum* W2 strain, showing a moderate activity, with an IC_50_ of 5.1 µM (Table 3), similar to that reported by Cui-Ying Ma et al. [70].

Chemical modifications to the structure of BA have produced compounds with better antiplasmodial activity. In fact, BA isolated from the crude extract of the leaves of *Pentalinon andrieuxii* (Apocynaceae) and their semisynthetic betulinic acid acetate (BAA) derivative were evaluated against *P. falciparum* F32 strain, and both demonstrated antimalarial activity, with IC_50_ values of 22.5 µM and 11.8 µM, respectively (Table 3). BAA had esterification of the C-3 hydroxyl group, which resulted in an improved activity [53]. In agreement, Cargnin et al. [79] observed that semisynthetic ursolic acid (UA) and BA derivatives modified at C-3 were shown to be more advantageous to antimalarial activity than simultaneous modifications at C-3 and C-28 positions, despite less evidenced, structural modifications at C-27 and C-28 positions also being reported as producing potentiation of the anti-*Plasmodium* activity [72].

Likewise, Sá and collaborators [29] observed potent in vitro anti-*P. falciparum* activity of BA and BAA derivative and found similar IC_50_ values: 9.89 µM and 5.99 µM, respectively (Table 3). However, the lethal dose effective to eliminate the parasite indicated LC_50_ values four- to six-fold higher than observed for parasite inhibition. Additionally, due to its in vitro activity, BAA was also assessed in vivo, at 10, 50, and 100 mg/kg doses by oral and intraperitoneal routes, in a *P. berghei*-infection model. The results demonstrated that treatment with BAA by oral route did not alter the levels of parasitemia in mice infected with *P. berghei* compared to mice treated with saline solution, and, by intraperitoneal route, BAA caused a dose-dependent reduction of parasitemia of at least 70% on the seventh day following infection, when compared to the vehicle-treated group [73]. 

In the search for effective bioactive hybrid molecules with improved properties compared to their parent compounds, Karagöz et al. [74] developed a series of betulinic acid/betulin-based dimer and hybrid compounds carrying ferrocene and/or artesunic acid moieties by de novo synthesis. These compounds were analyzed in vitro against *P. falciparum* 3D7 strain and it was found that a series of hybrids/dimers, betulinic acid/betulin, and artesunic acid hybrids showed the most potent antiplasmodial activities. In fact, the results revealed EC_50_ values of 0.085 µM for artesunic acid–betulinic acid hybrid, 0.0097 µM for artesunic acid, and 1.4 and 3.9 µM for BA and betulin, respectively (Table 3).

The molecular action mechanisms were also investigated in BA and derivatives against *P. falciparum* strains. Medeiros and collaborators [80] evaluated the in vitro antiplasmodial activity of imidazole derivatives of BA against *P. falciparum* NF54 and chloroquine-resistant isolated strains and observed that both derivatives presented good IC_50s_ values below 10 µM for both strains. In addition, the BA derivative with ester addition of butyric acid at C-3 also showed maturation inhibition of the parasite’s ring to schizont forms when compared to the start of the treatment, potentiating the antimalarial effect in inhibiting the parasite life cycle. In silico analysis presented a tight bind of BA derivative in the topoisomerase II-DNA complex, which suggests that BA forms a ligand–topoisomerase DNA complex by hydrogen bonding and hydrophobic interaction and blocks the cell replication. In addition, both UA and BA derivatives showed synergic effects when combined with artemisinin for both strains, while an additive interaction was observed when combined with chloroquine. Table 3 summarizes the main activity values of BA derived from extracts, and pure BA and its derivative compounds.

## 3. Concluding Remarks 

**Figure 3:**
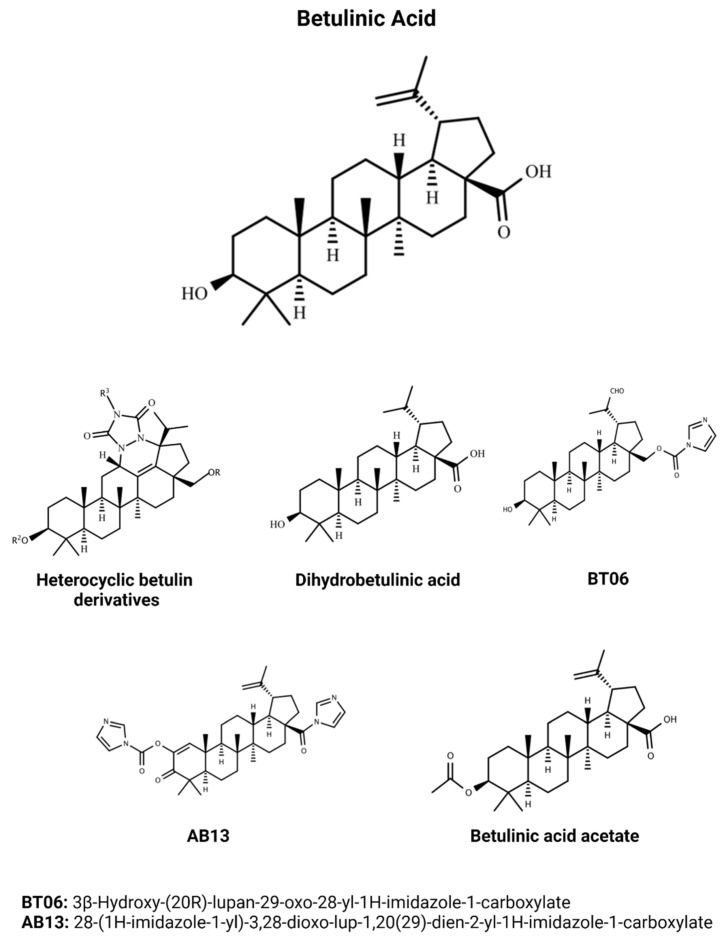
Chemical structures of the main efficacious BA and BA-derivative compounds which presented activity against *Leishmania*, *T. cruzi,* and *Plasmodium* in previous studies. BA structure (CID 64971) was obtained from PubChem [81].

BA is a triterpene compound which shares most of the biological activities related to this group of natural products. Regarding the development of new treatments to NTDs, it is well known that the biodiversity is an important source of molecules, and BA has pharmacological functions useful for application in the treatment of leishmaniasis, Chagas disease, and malaria. The most active compounds discussed in this review are showed in Figure 3.

In this work, we brought the most relevant literature data about the activity of BA and its derivatives against species and strains of Leishmania, *T. cruzi*, and *Plasmodium*. These data showed that extracts containing BA have low activity; however, the isolated compound (the most important to pharmacological development) presents an activity that needs to be considered by the research groups working in this field. The antiparasitic activity improves depending on the molecular changes of the triterpene backbone, as well as when a nanoparticle carrier is used, suggesting that pharmacokinetic and pharmacodynamic changes are needed during the development of a new drug based on BA or BA derivatives.

In addition, the dual effect of BA, the antiparasitic and immunomodulatory activities, may also be useful to treat infectious diseases caused by *Leishmania* and *Plasmodium*. For instance, the pathogenesis of localized cutaneous and mucocutaneous leishmaniasis lesions has the participation of the host’s immune response, in addition to the parasites, and the use of a drug showing this dual effect, such as BA, may contribute to the healing of the ulcer and reduction of treatment time and cost [55,56]. 

Similarly, in the cardiac form of the chronic phase of Chagas disease, the inflammatory process is responsible for the establishment of myocarditis, causing loss of cardiomyocytes and fibrosis deposition, ultimately leading to arrhythmias, heart failure, and death of Chagasic patients [18]. In this context, BA and its derivatives with dual effect might decrease inflammation and fibrosis in cardiac tissue without affecting parasite control, being an attractive alternative for the treatment of chronic Chagas cardiomyopathy [63].

During cerebral malaria (CM), a multi-factorial process is triggered, including sequestration, inflammation, and endothelial dysfunction in the microvasculature of the brain leading to coma. In this severe form of malaria, the inflammation outcomes depend on the delicate balance between proinflammatory and anti-inflammatory cytokines. Once there is a rupture of this balance, it may contribute to CM pathogenesis; therefore, therapeutic approaches could target cytokines and chemokines associated with CM severity. In this sense, triterpenes have been assessed in malaria experimentally and its antioxidant and pro-oxidant properties demonstrate a moderate phytotherapeutic effect for both decreasing parasitemia and alleviating inflammation [82,83,84].

The development and access of the population to new treatments are extremely important, mainly in poor countries and in places where parasite resistance is predominant. Based on the literature review, BA and its derivatives represent a group of compounds that need attention, since they can constitute an important set of compounds for treatment development against leishmaniasis, Chagas disease, and malaria, also in association with conventional medicines. Further studies are needed to help the scientific community to understand the pharmacodynamics and pharmacokinetics of BA and its derivatives, opening opportunities for clinical trials and drug development.

## Figures and Tables

**Table 1:** Summarized activity of betulinic acid against *Leishmania*.

Activity				
BA Source/Lupane Triterpenoids Derivatives	*Leishmania* Specie	Promastigotes	Amastigotes	Reference
*Pelliciera rhizophorae*	*L. donovani*	N.A.	N.A.	[41]
*Syzygium samarangense*	*L. major*	-	IC_50_ > 100 μM	[42]
*Millettia richardiana*	*L. major*	-	IC_50_ of 40 μM	[43]
Semisynthetic lupane triterpenoid 3b-Hydroxy-(20R)-lupan-29-oxo-28-yl-1H-imidazole-1- carboxylate	*L. infantum*	IC_50_ of 50.8 μM	-	[44]
Betulinic acid derivative 28-(1H-imidazole-1-yl)-3,28-dioxo-lup-1,20(29)-dien-2-yl-1H-imidazole-1-carboxylate	*L. infantum*	IC_50_ of 25.8 μM	-	[44]
Dihydrobetulinic acid	*L. donovani*	IC_50_ of 2.6 μM	IC_50_ of 4.1 μM	[45]
Betulonic acid	*L. donovani*	-	IC_50_ < 50 μM	[46]
Betulin 3-caffeate	*L. major*	IC_50_ > 100	-	[47]
Betulinic acid	*L. major*	IC_50_ of 2.6 μg/mL	-	[47]
Betulin aldehyde	*L. amazonensis*	IC_50_ > 300 μg/mL	-	[48]
Betulinic acid into nanoformulations containing nanochitosan	*L. major*	IC_50_ < 20 µg/mL	IC_50_ < 20 µg/mL	[49,50]
Betulinic acid into PLGA nanoparticles	*L. donovani*		Significantly reduce amastigote number in infected macrophages	[51]
BA5	*L. amazonensis*	IC_50_ of 4.5 ± 1.1 μM	IC_50_ of 4.1 ± 0.7 μM	[52]
	*L. major*	IC_50_ of 3.0 ± 0.8 μM		
	*L. braziliensis*	IC_50_ of 0.9 ± 1.1 μM		
	*L. infantum*	IC_50_ of 0.15 ± 0.05 μM		

N.A.: not active.

**Table 2:** Summarized activity of betulinic acid against *T. cruzi*.

Activity					
Source	Strain	Trypomastigotes	Epimastigotes	Amastigotes	Reference
*Bertholletia excelsa*	Y	100% at 500 µg/mL ^1^	-	-	[57]
		75.4% at 500 µg/mL ^2^	-	-	
BA	Tulahuen	-	IC_50_ of 50 µg/mL	-	[53]
			IC_50_ of 24.16 µg/mL		[59]
BA	Tulahuen		IC50 values of 73.43		[60]
Semi-synthetic derivative BA5	Y	IC_50_ of 1.8 µM		IC_50_ of 10.6 µM	[61]

N.A.: not active. ^1^ Acetone and methanol extract. ^2^ Hexane extract.

**Table 3:** Summarized activity of betulinic acid against *Plasmodium*.

Source	Strain	Activity	Reference
*Triphyophyllum peltatum*	NF54 (chloroquine-sensitive)	IC_50_ value of 10.46 µg/mL	[67]
*Ancistrocladus heyneanus*	NF54 (chloroquine-sensitive)	IC_50_ value of 10.46 µg/mL	[67]
*Uapaca nitida* Müll-Arg	T9-69 (chloroquine-sensitive)	IC_50_ of 19.6 µg/mL	[68]
	K1 (chloroquine-resistant)	IC_50_ of 25.9 µg/mL	[68]
*Gardenia saxatilis*	K1 (chloroquine-resistant)	IC_50s_ of 3.8 µg/mL and 2.9 µg/mL	[69]
*Diospyros quaesita* Thw	D6 (chloroquine-sensitive)	IC_50_ of 8.1 µM	[70]
	W2 (chloroquine-resistant)	IC_50_ of 8.3 µM	[70]
Pure BA	D6 (chloroquine-sensitive)	IC_50_ of 8.1 µM	[71]
	W2 (chloroquine-resistant)	IC_50_ of 8.3 µM	[71]
*Psorospermum glaberrimum*	W2 (chloroquine-resistant)	IC_50_ of 5.1 µM	[70]
*Pentalinon andrieuxii*	F32 (chloroquine-sensitive)	IC_50_ of 22.5 µM	[72]
Betulinic acid acetate (BAA)	F32 (chloroquine-sensitive)	IC_50_ of 11.8 µM	[57]
Pure BA	W2 (chloroquine-resistant)	IC_50_ of 9.89 µM	[73]
BAA	W2 (chloroquine-resistant)	IC_50_ of 5.99 µM	[73]
Artesunic acid–betulinic acid hybrid	3D7 (chloroquine-sensitive)	IC_50_ of 0.085 µM	[74]

## Data Availability

Data are contained within the article.

## References

[1] Neglected Tropical Diseases—GLOBAL. https://www.who.int/health-topics/neglected-tropical-diseases#tab=tab_2.

[2] Álvarez-Hernández D.-A., Rivero-Zambrano L., Martínez-Juárez L.-A., García-Rodríguez-Arana R. (2020). Overcoming the global burden of neglected tropical diseases. Ther. Adv. Infect. Dis..

[3] De Souza D.K., Picado A., Biéler S., Nogaro S., Ndung’U J.M. (2020). Diagnosis of neglected tropical diseases during and after the COVID-19 pandemic. PLoS Negl. Trop. Dis..

[4] Tilli M., Olliaro P., Gobbi F., Bisoffi Z., Bartoloni A., Zammarchi L. (2021). Neglected tropical diseases in non-endemic countries in the era of COVID-19 pandemic: The great forgotten. J. Travel Med..

[5] Ehrenberg J.P., Utzinger J., Fontes G., da Rocha E.M.M., Ehrenberg N., Zhou X.-N., Steinmann P. (2021). Efforts to mitigate the economic impact of the COVID-19 pandemic: Potential entry points for neglected tropical diseases. Infect. Dis. Poverty.

[6] Nargesi S., Bongomin F., Hedayati M.T. (2021). The impact of COVID-19 pandemic on AIDS-related mycoses and fungal neglected tropical diseases: Why should we worry?. PLoS Negl. Trop. Dis..

[7] Mitra A.K., Mawson A.R. (2017). Neglected Tropical Diseases: Epidemiology and Global Burden. Trop. Med. Infect. Dis..

[8] Bhattacharya A., Corbeil A., do Monte-Neto R.L., Fernandez-Prada C. (2020). Of Drugs and Trypanosomatids: New Tools and Knowledge to Reduce Bottlenecks in Drug Discovery. Genes.

[9] Reithinger R., Dujardin J.-C., Louzir H., Pirmez C., Alexander B., Brooker S. (2007). Cutaneous leishmaniasis. Lancet Infect. Dis..

[10] Alvar J., Vélez I.D., Bern C., Herrero M., Desjeux P., Cano J., Jannin J., den Boer M., the WHO Leishmaniasis Control Team (2012). Leishmaniasis Worldwide and Global Estimates of Its Incidence. PLoS ONE.

[11] Kassi M., Kassi M., Afghan A.K., Rehman R., Kasi P.M. (2008). Marring Leishmaniasis: The Stigmatization and the Impact of Cutaneous Leishmaniasis in Pakistan and Afghanistan. PLoS Negl. Trop. Dis..

[12] Garcia E., Azambuja P. (1991). Development and interactions of *Trypanosoma cruzi* within the insect vector. Parasitol. Today.

[13] Yoshida N. (2008). *Trypanosoma cruzi* infection by oral route: How the interplay between parasite and host components modulates infectivity. Parasitol. Int..

[14] Losada Galván I., Alonso-Padilla J., Cortés-Serra N., Alonso-Vega C., Gascón J., Pinazo M.J. (2021). Benznidazole for the Treatment of Chagas Disease. Expert Rev. anti-Infect. Ther..

[15] Mills R.M. (2020). Chagas Disease: Epidemiology and Barriers to Treatment. Am. J. Med..

[16] Moxon C.A., Gibbins M., McGuinness D., Milner D.A., Marti M. (2020). New Insights into Malaria Pathogenesis. Annu. Rev. Pathol. Mech. Dis..

[17] Milner D.A. (2017). Malaria Pathogenesis. Cold Spring Harb. Perspect. Med..

[18] Sato S. (2021). Plasmodium—A brief introduction to the parasites causing human malaria and their basic biology. J. Physiol. Anthropol..

[19] World Malaria Report 2021. https://www.who.int/publications-detail-redirect/9789240040496.

[20] Maier A.G., Matuschewski K., Zhang M., Rug M. (2019). Plasmodium Falciparum. Trends Parasitol..

[21] Ashley E.A., Phyo A.P. (2018). Drugs in Development for Malaria. Drugs.

[22] Nevagi R.J., Good M.F., Stanisic D.I. (2021). Plasmodium infection and drug cure for malaria vaccine development. Expert Rev. Vaccines.

[23] Laurens M.B. (2019). RTS,S/AS01 vaccine (Mosquirix™): An overview. Hum. Vaccines Immunother..

[24] Roatt B.M., de Oliveira Cardoso J.M., De Brito R.C.F., Coura-Vital W., de Oliveira Aguiar-Soares R.D., Reis A.B. (2020). Recent advances and new strategies on leishmaniasis treatment. Appl. Microbiol. Biotechnol..

[25] Sinha P.K., Roddy P., Palma P.P., Kociejowski A., Lima M.A., Das V.N.R., Gupta J., Kumar N., Mitra G., Saint-Sauveur J.-F. (2010). Effectiveness and Safety of Liposomal Amphotericin B for Visceral Leishmaniasis under Routine Program Conditions in Bihar, India. Am. J. Trop. Med. Hyg..

[26] Marin-Neto J.A., Simões M.V., Junior A.R. (2013). Pathogenesis of chronic Chagas cardiomyopathy: The role of coronary microvascular derangements. Rev. Soc. Bras. Med. Trop..

[27] Viotti R., Vigliano C., Lococo B., Bertocchi G., Petti M., Alvarez M.G., Postan M., Armenti A. (2006). Long-Term Cardiac Outcomes of Treating Chronic Chagas Disease with Benznidazole versus No Treatment: A Nonrandomized Trial. Ann. Intern. Med..

[28] Antonio Marin-Neto J., Rassi A., Avezum A., Mattos A.C., Rassi A. (2009). The BENEFIT Trial: Testing the Hypothesis That Trypanocidal Therapy Is Beneficial for Patients with Chronic Chagas Heart Disease. Mem. Inst. Oswaldo Cruz.

[29] Wicht K.J., Mok S., Fidock D.A. (2020). Molecular Mechanisms of Drug Resistance in Plasmodium Falciparum Malaria. Annu. Rev. Microbiol..

[30] Burrows J.N., Burlot E., Campo B., Cherbuin S., Jeanneret S., Leroy D., Spangenberg T., Waterson D., Wells T.N., Willis P. (2014). Antimalarial Drug Discovery - the Path towards Eradication. Parasitology.

[31] Achan J., Talisuna A.O., Erhart A., Yeka A., Tibenderana J.K., Baliraine F.N., Rosenthal P.J., D’Alessandro U. (2011). Quinine, an Old Anti-Malarial Drug in a Modern World: Role in the Treatment of Malaria. Malar. J..

[32] Eastman R.T., Fidock D.A. (2009). Artemisinin-Based Combination Therapies: A Vital Tool in Efforts to Eliminate Malaria. Nat. Rev. Microbiol..

[33] Kayser O., Kiderlen A.F., Croft S.L. (2003). Natural Products as Antiparasitic Drugs. Parasitol. Res..

[34] Naula C., Parsons M., Mottram J.C. (2005). Protein Kinases as Drug Targets in Trypanosomes and Leishmania. Biochim. Biophys. Acta.

[35] Späth G.F., Clos J. (2016). Joining Forces: First Application of a Rapamycin-Induced Dimerizable Cre System for Conditional Null Mutant Analysis in Leishmania. Mol. Microbiol..

[36] Santos E.D.S., Silva D.K.C., dos Reis B.P.Z.C., Barreto B.C., Cardoso C.M.A., dos Santos R.R., Meira C.S., Soares M.B.P. (2021). Immunomodulation for the Treatment of Chronic Chagas Disease Cardiomyopathy: A New Approach to an Old Enemy. Front. Cell. Infect. Microbiol..

[37] Barrow P., Dujardin J.C., Fasel N., Greenwood A.D., Osterrieder K., Lomonossoff G., Fiori P.L., Atterbury R., Rossi M., Lalle M. (2020). Viruses of protozoan parasites and viral therapy: Is the time now right?. Virol. J..

[38] Altamura F., Rajesh R., Catta-Preta C.M.C., Moretti N.S., Cestari I. (2020). The current drug discovery landscape for trypanosomiasis and leishmaniasis: Challenges and strategies to identify drug targets. Drug Dev. Res..

[39] Hordyjewska A., Ostapiuk A., Horecka A., Kurzepa J. (2019). Betulin and betulinic acid: Triterpenoids derivatives with a powerful biological potential. Phytochem. Rev..

[40] Ríos J.-L. (2010). Effects of triterpenes on the immune system. J. Ethnopharmacol..

[41] López D., Cherigo L., Spadafora C., Lozamejia M.A., Martínez-Luis S. (2015). Phytochemical composition, antiparasitic and α–glucosidase inhibition activities from *Pelliciera rhizophorae*. Chem. Cent. J..

[42] Samy M.N., Mamdouh N.S., Sugimoto S., Matsunami K., Otsuka H., Kamel M.S. (2014). Taxiphyllin 6′-O-Gallate, Actinidioionoside 6′-O-Gallate and Myricetrin 2″-O-Sulfate from the Leaves of Syzygium Samarangense and Their Biological Activities. Chem. Pharm. Bull..

[43] Rajemiarimiraho M., Banzouzi J.-T., Nicolau-Travers M.-L., Ramos S., Ali Z., Bories C., Rakotonandrasana O.L., Rakotonandrasana S., Andrianary P.A., Benoit-Vical F. (2014). Antiprotozoal Activities of *Millettia richardiana* (Fabaceae) from Madagascar. Molecules.

[44] Sousa M.C., Varandas R., Santos R.C., Santos-Rosa M., Alves V., Salvador J.A.R. (2014). Antileishmanial Activity of Semisynthetic Lupane Triterpenoids Betulin and Betulinic Acid Derivatives: Synergistic Effects with Miltefosine. PLoS ONE.

[45] Chowdhury A.R., Mandal S., Goswami A., Ghosh M., Mandal L., Chakraborty D., Ganguly A., Tripathi G., Mukhopadhyay S., Bandyopadhyay S. (2003). Dihydrobetulinic Acid Induces Apoptosis in Leishmania donovani by Targeting DNA Topoisomerase I and II: Implications in Antileishmanial Therapy. Mol. Med..

[46] Alakurtti S., Bergström P., Sacerdoti-Sierra N., Jaffe C.L., Yli-Kauhaluoma J. (2010). Anti-leishmanial activity of betulin derivatives. J. Antibiot..

[47] Takahashi M., Fuchino H., Sekita S., Satake M. (2004). In vitro leishmanicidal activity of some scarce natural products. Phytother. Res..

[48] Gantier J.-C. (1996). Isolation of Leishmanicidal Triterpenes and Lignans from the Amazonian Liana *Doliocarpus dentatus* (Dilleniaceae). Phytother. Res..

[49] Zadeh Mehrizi T., Shafiee Ardestani M., Haji Molla Hoseini M., Khamesipour A., Mosaffa N., Ramezani A. (2018). Novel Nanosized Chitosan-Betulinic Acid Against Resistant Leishmania Major and First Clinical Observation of Such Parasite in Kidney. Sci. Rep..

[50] Mehrizi T.Z., Khamesipour A., Ardestani M.S., Shahmabadi H.E., Hoseini M.H.M., Mosaffa N., Ramezani A. (2019). Comparative analysis between four model nanoformulations of amphotericin B-chitosan, amphotericin B-dendrimer, betulinic acid-chitosan and betulinic acid-dendrimer for treatment of *Leishmania major*: Real-time PCR assay plus. Int. J. Nanomed..

[51] Halder A., Shukla D., Das S., Roy P., Mukherjee A., Saha B. (2018). Lactoferrin-modified Betulinic Acid-loaded PLGA nanoparticles are strong anti-leishmanials. Cytokine.

[52] Magalhães T.B.D.S., Silva D., keyse C., Teixeira J.D.S., de Lima J.D.T., Barbosa-Filho J.M., Moreira D.R.M., Guimarães E.T., Soares M.B.P. (2014). BA5, a Betulinic Acid Derivative, Induces G0/G1 Cell Arrest, Apoptosis Like-Death, and Morphological Alterations in *Leishmania* sp.. Front. Pharmacol..

[53] Domínguez-Carmona D., Escalante-Erosa F., García-Sosa K., Ruiz-Pinell G., Gutierrez-Yapu D., Chan-Bacab M., Giménez-Turba A., Peña-Rodríguez L. (2010). Antiprotozoal activity of Betulinic acid derivatives. Phytomed. Int. J. Phytother. Phytopharm..

[54] Renda G., Gökkaya I., Şöhretoğlu D. (2021). Immunomodulatory properties of triterpenes. Phytochem. Rev..

[55] Volpedo G., Pacheco-Fernandez T., Holcomb E.A., Cipriano N., Cox B., Satoskar A.R. (2021). Mechanisms of Immunopathogenesis in Cutaneous Leishmaniasis And Post Kala-azar Dermal Leishmaniasis (PKDL). Front. Cell. Infect. Microbiol..

[56] Scorza B.M., Carvalho E.M., Wilson M.E. (2017). Cutaneous Manifestations of Human and Murine Leishmaniasis. Int. J. Mol. Sci..

[57] Campos F., Januário A.H., Rosas L.V., Nascimento S.K., Pereira P.S., Franca S., Cordeiro M.S., Toldo M.P., de Albuquerque S. (2005). Trypanocidal activity of extracts and fractions of *Bertholletia excelsa*. Fitoterapia.

[58] Rosas L., Cordeiro M., Campos F., Nascimento S., Januário A., França S., Nomizo A., Toldo M., Albuquerque S., Pereira P. (2007). In vitro evaluation of the cytotoxic and trypanocidal activities of *Ampelozizyphus amazonicus* (Rhamnaceae). Braz. J. Med. Biol. Res..

[59] Cretton S., Bréant L., Pourrez L., Ambuehl C., Perozzo R., Marcourt L., Kaiser M., Cuendet M., Christen P. (2015). Chemical constituents from Waltheria indica exert in vitro activity against *Trypanosoma brucei* and *T. cruzi*. Fitoterapia.

[60] Sousa P.L., Souza R.O.D.S., Tessarolo L.D., Menezes R., Sampaio T.L., Canuto J.A., Martins A.M.C. (2017). Betulinic acid induces cell death by necrosis in *Trypanosoma cruzi*. Acta Trop..

[61] Meira C.S., Filho J.M.B., Lanfredi-Rangel A., Guimarães E.T., Moreira D.R., Soares M.B.P. (2016). Antiparasitic evaluation of betulinic acid derivatives reveals effective and selective anti-*Trypanosoma cruzi* inhibitors. Exp. Parasitol..

[62] Portella D.C.N., Rossi E.A., Paredes B.D., Bastos T.M., Meira C.S., Nonaka C.V.K., Silva D.N., Improta-Caria A., Moreira D.R.M., Leite A.C.L. (2021). A Novel High-Content Screening-Based Method for Anti-*Trypanosoma cruzi* Drug Discovery Using Human-Induced Pluripotent Stem Cell-Derived Cardiomyocytes. Stem Cells Int..

[63] Alirol E., Schrumpf D., Heradi J.A., Riedel A., De Patoul C., Quere M., Chappuis F. (2012). Nifurtimox-Eflornithine Combination Therapy for Second-Stage Gambiense Human African Trypanosomiasis: Médecins Sans Frontières Experience in the Democratic Republic of the Congo. Clin. Infect. Dis. Off. Publ. Infect. Dis. Soc. Am..

[64] Diniz L., Urbina J.A., De Andrade I.M., Mazzeti A.L., Martins T.A.F., Caldas I., Talvani A., Ribeiro I., Bahia M.T. (2013). Benznidazole and Posaconazole in Experimental Chagas Disease: Positive Interaction in Concomitant and Sequential Treatments. PLoS Negl. Trop. Dis..

[65] Meira C.S., Santos E.D.S., Espírito-Santo R.F.D., Vasconcelos J., Orge I., Nonaka C.K.V., Barreto B.C., Caria A.C.I., Silva D., Filho J.M.B. (2019). Betulinic Acid Derivative BA5, Attenuates Inflammation and Fibrosis in Experimental Chronic Chagas Disease Cardiomyopathy by Inducing IL-10 and M2 Polarization. Front. Immunol..

[66] Da Silva G.N., Maria N.R., Schuck D.C., Cruz L.N., de Moraes M.S., Nakabashi M., Graebin C., Gosmann G., Garcia C.R., Gnoatto S.C. (2013). Two series of new semisynthetic triterpene derivatives: Differences in anti-malarial activity, cytotoxicity and mechanism of action. Malar. J..

[67] Bringmann G., Saeb W., Assi L., François G., Narayanan A.S.S., Peters K., Peters E.-M. (1997). Betulinic Acid: Isolation from *Triphyophyllum peltatum* and *Ancistrocladus heyneanus*, Antimalarial Activity, and Crystal Structure of the Benzyl Ester. Planta Med..

[68] Steele J.C.P., Warhurst D.C., Kirby G.C., Simmonds M.S.J. (1999). In vitro and In vivo evaluation of betulinic acid as an antimalarial. Phytother. Res..

[69] Suksamrarn A., Tanachatchairatana T., Kanokmedhakul S. (2003). Antiplasmodial triterpenes from twigs of *Gardenia saxatilis*. J. Ethnopharmacol..

[70] Ma C.-Y., Musoke S.F., Tan G.T., Sydara K., Bouamanivong S., Southavong B., Soejarto D.D., Fong H.H.S., Zhang H.-J. (2008). Study of Antimalarial Activity of Chemical Constituents fromDiospyros quaesita. Chem. Biodivers..

[71] Ndjakou Lenta B., Devkota K.P., Ngouela S., Fekam Boyom F., Naz Q., Choudhary M.I., Tsamo E., Rosenthal P.J., Sewald N. (2008). Anti-Plasmodial and Cholinesterase Inhibiting Activities of Some Constituents of Psorospermum Glaberrimum. Chem. Pharm. Bull..

[72] Isah M., Ibrahim M.A., Mohammed A., Aliyu A.B., Masola B., Coetzer T. (2016). A systematic review of pentacyclic triterpenes and their derivatives as chemotherapeutic agents against tropical parasitic diseases. Parasitology.

[73] de Sá M.S., Costa J.F.O., Krettli A.U., Zalis M.G., Maia G.L.D.A., Sette I.M.F., Câmara C.D.A., Filho J.M.B., Giulietti-Harley A.M., Ribeiro Dos Santos R. (2009). Antimalarial Activity of Betulinic Acid and Derivatives in Vitro against Plasmodium Falciparum and in Vivo in P. Berghei-Infected Mice. Parasitol. Res..

[74] Karagöz A.Ç., Leidenberger M., Hahn F., Hampel F., Friedrich O., Marschall M., Kappes B., Tsogoeva S.B. (2019). Synthesis of New Betulinic Acid/Betulin-Derived Dimers and Hybrids with Potent Antimalarial and Antiviral Activities. Bioorganic Med. Chem..

[75] Ziegler H.L., Staalsø T., Jaroszewski J.W. (2006). Loading of Erythrocyte Membrane with Pentacyclic Triterpenes Inhibits *Plasmodium falciparum* Invasion. Planta Med..

[76] Friedrichson T., Kurzchalia T.V. (1998). Microdomains of GPI-anchored proteins in living cells revealed by crosslinking. Nature.

[77] Dubinin M.V., Semenova A.A., Ilzorkina A.I., Mikheeva I.B., Yashin V.A., Penkov N.V., Vydrina V.A., Ishmuratov G.Y., Sharapov V.A., Khoroshavina E.I. (2020). Effect of betulin and betulonic acid on isolated rat liver mitochondria and liposomes. Biochim. Biophys. Acta Biomembr..

[78] Silva G.N.S., Schuck D.C., Cruz L.N., Moraes M.S., Nakabashi M., Gosmann G., Garcia C.R.S., Gnoatto S.C.B. (2014). Investigation of antimalarial activity, cytotoxicity and action mechanism of piperazine derivatives of betulinic acid. Trop. Med. Int. Health.

[79] Cargnin S.T., Staudt A.F., Medeiros P., de Medeiros Sol Sol D., de Azevedo Dos Santos A.P., Zanchi F.B., Gosmann G., Puyet A., Garcia Teles C.B., Gnoatto S.B. (2018). Semisynthesis, Cytotoxicity, Antimalarial Evaluation and Structure-Activity Relationship of Two Series of Triterpene Derivatives. Bioorganic Med. Chem. Lett..

[80] Sol Sol de Medeiros D., Tasca Cargnin S., Azevedo Dos Santos A.P., de Souza Rodrigues M., Berton Zanchi F., Soares de Maria de Medeiros P., de Almeida E Silva A., Bioni Garcia Teles C., Baggio Gnoatto S.C. (2021). Ursolic and Betulinic Semisynthetic Derivatives Show Activity against CQ-Resistant Plasmodium Falciparum Isolated from Amazonia. Chem. Biol. Drug Des..

[81] Betulinic Acid | C30H48O3—PubChem. https://pubchem.ncbi.nlm.nih.gov/compound/Betulinic-acid#section=2D-Structure.

[82] Mavondo G.A., Mkhwanazi B.N., Mzingwane M.L., Dangarembizi R., Zambuko B., Moyo O., Musiwaro P., Chikuse F.F., Rakabopa C., Mpofu T. (2019). Malarial Inflammation-Driven Pathophysiology and Its Attenuation by Triterpene Phytotherapeutics. Parasitology and Microbiology Research.

[83] Storm J., Craig A. (2014). Pathogenesis of cerebral malaria—Inflammation and cytoadherence. Front. Cell. Infect. Microbiol..

[84] Dunst J., Kamena F., Matuschewski K. (2017). Cytokines and Chemokines in Cerebral Malaria Pathogenesis. Front. Cell. Infect. Microbiol..

